# Follow up after sample size re-estimation in a breast cancer randomized trial for disease-free survival

**DOI:** 10.1186/s13063-019-3632-9

**Published:** 2019-08-23

**Authors:** Erinn M. Hade, Gregory S. Young, Richard R. Love

**Affiliations:** 1 Department of Biomedical Informatics, Center for Biostatistics, College of Medicine, The Ohio State University, 1800 Cannon Drive, 320 Lincoln Tower, Columbus, OH 43210 USA; 2 Department of Mathematics, Statistics, and Computer Science, Marquette University, Milwaukee, WI USA

**Keywords:** Blinded sample size re-estimation, Breast cancer, Time to event, Control group failure

## Abstract

**Background:**

While the clinical trials and statistical methodology literature on sample size re-estimation (SSRE) is robust, evaluation of SSRE procedures following the completion of a clinical trial has been sparsely reported. In blinded sample size re-estimation, only nuisance parameters are re-estimated, and the blinding of the current trial treatment effect is preserved. Blinded re-estimation procedures are well-accepted by regulatory agencies and funders. We review our experience of sample size re-estimation in a large international, National Institutes of Health funded clinical trial for adjuvant breast cancer treatment, and evaluate our blinded sample size re-estimation procedure for this time-to-event trial. We evaluated the SSRE procedure by examining assumptions made during the re-estimation process, estimates resulting from re-estimation, and the impact on final trial results with and without the addition of participants, following sample size re-estimation.

**Methods:**

We compared the control group failure probabilities estimated at the time of SSRE to estimates used in the original planning, to the final un-blinded control group failure probability estimates for those included in the SSRE procedure (SSRE cohort), and to the final total control group failure probability estimates. The impact of re-estimation on the final comparison between randomized treatment groups is evaluated for those in the originally planned cohort (*n* = 340) and for the combination of those recruited in the originally planned cohort and those added after re-estimation (*n* = 509).

**Results:**

Very little difference is observed between the originally planned cohort and all randomized patients in the control group failure probabilities over time or in the overall hazard ratio estimating treatment effect (originally planned cohort HR 1.25 (0.86, 1.79); all randomized cohort HR 1.24 95% CI (0.91, 1.68)). At the time of blinded SSRE, the estimated control group failure probabilities at 3 years (0.24) and 5 years (0.40) were similar to those for the SSRE cohort once un-blinded (3 years, 0.22 (0.16, 0.30); 5 years, 0.33 (0.26, 0.41)).

**Conclusions:**

We found that our re-estimation procedure performed reasonably well in estimating the control group failure probabilities at the time of re-estimation. Particularly for time-to-event outcomes, pre-planned blinded SSRE procedures may be the best option to aid in maintaining power.

**Trial registration:**

ClinicalTrials.gov, NCT00201851. Registered on 9 September 2005. Retrospectively registered.

## Background

Evaluation of sample size re-estimation procedures, following the completion of a clinical trial, has been sparsely reported in the literature [1, 2]. Based on the acknowledgment of uncertainty in the information used as a basis for sample size determination at the design stage, various methods of re-evaluation of sample size have been developed [3–8]. Sample size re-estimation (SSRE) methods have evolved during the past 20 years, and take several general forms. Broadly grouped, re-estimation procedures can be un-blinded, those that utilize the un-blinded current trial data including the estimated treatment effect estimate. Un-blinded sample size re-estimation may be accompanied by interim monitoring for futility or efficacy. Several have compared the merits of un-blinded sample size re-estimation procedures and those of traditional group sequential methods [9–12]. In blinded sample size re-estimation, only nuisance parameters are re-estimated, and the blinding of the current trial treatment effect is preserved. These nuisance parameters may include the standard deviation in the continuous outcome setting, or the control group failure probabilities in the survival (time to event) context. Blinded re-estimation procedures are well-accepted by regulatory agencies and funders. This class of re-estimation procedures has shown no evidence of inflating type one error and clearly maintains study integrity [13, 14].

However, strictly blinded, sample size re-estimation methods may increase sample sizes when the test treatment is futile. Partially blinded procedures have been proposed as an alternative to fully blinded procedures [14, 15]; these may un-blind the treatment allocation to the Data and Safety Monitoring Committee (DSMC), but would only use the nuisance parameter estimates in the re-estimation, not the estimated treatment effect. Both the partially and fully blinded procedures may also incorporate data external to the trial as well as information from the combined treatment groups. These procedures are often discussed as one of the many adaptations available to methodologists in the design and implementation of an efficient trial.

The 2010 draft guidance from the US Food and Drug Administration provides review and recommendations for adaptive clinical trial designs, and implications of these in the regulatory setting [16]. While SSRE methods have been discussed widely, implementation of these has not been widespread, particularly among non-pharmaceutical-sponsored studies [2] and reporting on them is even more rare [1]. Empiric evaluation of SSRE methods is necessary to more fully understand their merits and limitations, and to refine these procedures for more efficient design.

During the course of a large, multi-site, international clinical trial funded by the National Institutes of Health, we re-evaluated the required sample size near the end of the planned accrual period using a blinded SSRE procedure [13, 17]. Herein, we review our SSRE experience, consider implications of SSRE in the primary analysis, and examine the control-group failure probabilities in those who contributed information to the SSRE procedure (the SSRE cohort) and in all participants recruited to the control group, to see how well our re-estimation procedure performed. Further, we described the hazard functions by randomized group to explore if they converged dramatically during the course of follow up. Our goal is to provide empirical information on the use of SSRE as a means to improve reporting and transparency in its use.

### Adjuvant trial of timing of surgical oophorectomy

Initiated in 2003, recruitment to a randomized, international, breast cancer trial, which was studying the effects of surgical timing during the menstrual phase on disease-free survival in women with hormone receptor positive breast cancer, was expanded through a blinded SSRE procedure. The details of this process, the rationale of the SSRE methodology, and the primary trial results have been detailed elsewhere (ClinicalTrials.gov number 00201851) [13, 17]. This trial recruited premenopausal women with hormone receptor positive, operable breast cancer in the Philippines, Vietnam, and Morocco, and compared how the timing of surgical oophorectomy and mastectomy affected time to recurrence [17]. A significant body of prior work had suggested that adjuvant oophorectomy surgery during the luteal phase of the menstrual cycle may provide longer disease-free and overall survival as compared to surgery in the follicular phase of the menstrual cycle [18–21]. As follow up information on the overall failure probabilities (of recurrence or death) for all randomized patients was being monitored, concern arose that the failure probabilities assumed for the control group were too high during the planning stage [19]. Given these overall probabilities and mature data from a prior trial, the failure probabilities used at the design stage appeared too high resulting in a loss of power for the hazard ratio of interest within a reasonable period of trial accrual and follow up. Extending follow up to obtain the number of events expected during the initial planning was not a viable option due to evidence in several previous trials of converging hazard functions; this evidence was apparent only after the initial trial planning. Therefore, we proceeded to develop a method to re-estimate sample size in this time-to-event trial based on the blinded trial data. In cooperation with our DSMC, we agreed to implement a straightforward blinded re-estimation framework for time to event outcomes [13]. Given that this SSRE was un-planned at the design stage, for an unusual intervention, a blinded procedure was critical to maintaining the overall study integrity and confidence of funders and reviewers of the study. The trial team and DSMC were eager to have no true or perceived lapses in conduct or on the trial design. Moreover, they did not want to risk the perception that the sample size was manipulated because a smaller effect than was anticipated was found. The blinded SSRE procedure utilizes the current blinded (overall) trial failure-time data and either previous data external to the trial, with long-term follow up or simulated data with long-term follow-up to supplement the survival experience past when would have been observed in the current trial.

Blinded SSRE was implemented toward the end of the originally planned accrual period. We utilized data from a previous trial in a similar patient population receiving the same therapy (but who were not randomized by timing of surgery) and the current blinded trial data, to estimate the control-group failure probabilities, and subsequently sample size. Estimates from our re-estimation procedure (that incorporated previous preliminary data and the current blinded trial data) indicated that the control failure probabilities were compatible with what was proposed at the time of the study design and the distribution of bootstrap re-estimates of sample size indicated no increase was needed for the proposed hazard ratio (HR).

## Methods

The initial study design planned to randomize 340 women in equal proportions to either immediate surgery in the next 1–6 days, which was expected to be in the follicular phase of the menstrual cycle, or to scheduled surgery during the next mid-luteal phase of the menstrual cycle. The original design assumed a final primary analysis by log-rank test would have 80% power, with 5% two-sided type one error, to detect a HR of 0.58 in favor of scheduled surgery, with 2–3% loss to follow up. This design required 113 events, with accrual time of 2 years and 4 additional years of follow up. However, the effect estimate used for the proposed sample size, was felt to be optimistic based on more current comparisons in data and sources external to the trial. The sample size was increased by 170 patients, to a total of 510 randomized (with a required 175 total number of events), in order to target a proposed HR of 0.65. Final follow up was completed in 2013 and the results of the primary (disease-free survival) and secondary (overall survival) trial endpoints have been previously published, including the trial Consolidated Standards of Reporting Trials (CONSORT) diagram [17]. Since our SSRE procedure was blinded, in the current work we aimed to examine assumptions made during the re-estimation process, the estimates made in the re-estimation procedure, and the impact on final trial results with and without the addition of participants (the original study cohort size).

Given that estimation of the control failure function over the course of the follow up period is critical to re-estimation of sample size in time-to-event studies, we explored estimation of the failure probabilities at 3 and 5 years after surgical treatment, for disease-free survival [22]. In particular we compared the control group (immediate/follicular-phase surgery) failure probabilities estimated at the time of SSRE to (1) those originally used in planning the trial, (2) the final control-group failure probabilities at the completion of the trial in the SSRE cohort (those participants in the current trial with information included in the SSRE procedure), and (3) the control group failure probabilities estimated for all control-group participants, including those in the originally planned cohort (the first 340 randomized patients) and those in the expansion cohort (the 170 patients added after re-estimation).

### Statistical methods

To complement these summaries of participants’ failure experience over time, we estimated the hazard functions in the initial and expansion cohorts and more finely, by major recruitment site, to describe changes throughout the follow up period. These estimates emphasize the instability typical of failure estimates at the interim time in trials when SSRE procedures are performed. Failure probabilities were calculated though the method of Kaplan and Meier [23]. Smoothed hazards were estimated via a kernel smoothing method with Epanechnikov kernel [24]. The impact of re-estimation on the final inference between randomized treatment groups was evaluated by estimating the hazard ratio (95% confidence interval) for those originally planned to be enrolled and for the combination of those recruited in the originally planned cohort and those added after re-estimation. The event rate, the number of events per 1000 person years was calculated in each treatment group, and the hazard ratio was estimated by Cox proportional hazard regression. Data management, sample size estimation, and bootstrap re-sampling were performed using SAS software, Version 9.1.3 and 9.4 of the SAS System Copyright 2003, 2012 SAS Institute Inc. Further analysis and hazard estimation were performed using STATA Statistical Software, version 13 (StataCorp. 2013. *Stata Statistical Software: Release 13*. College Station, TX USA: StataCorp LP.)

## Results

Table 1 describes the failure probability distribution over time, the average follow up time, the event rate per 1000 person years, and the HR for the originally planned set of patients, and for all randomized patients accrued to the trial. Very little difference was observed in the failure function over time or in the overall HR estimating treatment benefit between the originally planned cohort and all randomized patients (final HR (95% CI) 1.24 (0.91, 1.68); for the original study cohort, HR 1.25 (0.86, 1.79)). However, the width of the confidence interval is approximately 17% narrower with the larger sample size.

**Table 1:** Disease-free failure estimates, follow up time and hazard ratios

	All randomized patients^a^				Original cohort of randomized patients^a^			
	Immediate/follicular phase		Scheduled/luteal phase		Immediate/follicular phase		Scheduled/luteal phase	
Total randomized	255		244		167		167	
Year	Total (Failures)	Failure probability (95% CI)	Total (Failures)	Failure probability (95% CI)	Total (Failures)	Failure probability (95% CI)	Total (Failures)	Failure probability (95% CI)
1	246 (10)	0.04 (0.02, 0.07)	226 (18)	0.07 (0.05, 0.11)	163 (5)	0.03 (0.01, 0.07)	157 (11)	0.07 (0.4, 0.12)
3	210 (36)	0.18 (0.14, 0.23)	178 (45)	0.26 (0.21, 0.32)	137 (26)	0.19 (0.13, 0.25)	124 (31)	0.25 (0.19, 0.33)
5	118 (26)	0.29 (0.24, 0.36)	102 (20)	0.36 (0.30, 0.43)	116 (17)	0.29 (0.23, 0.36)	102 (18)	0.36 (0.29, 0.44)
Total events^b^	78		85		54		62	
Average follow up time, *years* (SD)	4.41(1.5)		4.08 (1.8)		4.77 (1.6)		4.42 (1.9)	
Event rate^b^	69.4		85.4		67.8		83.9	
HR^b^ (95% CI)	1.24 (0.91, 1.68)				1.25 (0.86, 1.79)			

^a^ A total of 499 of 509 of all randomized patients and 334 of 340 original-cohort patients were evaluable. Of the initial cohort, 278 were recruited at the time of sample size re-estimation (SSRE)

^b^ Per 1000 person years; through 6 years of follow up

At the time of sample size re-estimation, our procedure estimated that the failure probabilities at 3 and 5 years of follow up in the immediate/follicular-phase-surgery group were close to those planned at time of the trial design (Table 2). Recall that the failure probabilities estimated at SSRE utilized the blinded trial data up until the time of SSRE, and therefore follow up time and the number of events from the current trial were limited. Consequently, the blinded trial data were augmented by data from a previous trial with longer follow up time, which had informed the original design. The decision rule for the SSRE indicated choice of the re-estimated sample size at the upper 80th percentile of the re-estimated sample size bootstrap distribution. The failure probabilities corresponding to this sample size were 0.24 at year 3 and 0.40 at year 5, approximately the 20th percentile of the re-estimated failure probability bootstrap distribution. When this re-estimation cohort was followed until trial close, failure probabilities were slightly lower than those estimated at the time of sample size re-estimation, but within our confidence limits (at 3 years, 0.22 (0.16, 0.30); at 5 years, 0.33 (0.26, 0.41)). The final failure rate for all control-group randomized patients was somewhat smaller than for those estimated at the time of sample size re-estimation (at 3 years, 0.18 (0.14, 0.23); at 5 years, 0.29 (0.24, 0.36)).

**Table 2:** Disease-free failure probability estimates from design, SSRE, and final analysis stages in the control group (immediate/follicular-phase-surgery) (gray line in Figs. 1, 2 and 3)

Time (years)	Prior trial data	Estimates from trial design	Estimated by SSRE procedure^a^		Final for SSRE cohort (95% CI)	Final for all randomized (95% CI)
			Median (5%, 95%)	Estimates used in SSRE^b^		
3	0.16	0.28	0.30 (0.22, 0.39)	0.24	0.22 (0.16, 0.30)	0.18 (0.14, 0.23)
5	0.26	0.40	0.49 (0.35, 0.67)	0.40	0.33 (0.26, 0.41)	0.29 (0.24, 0.36)

^a^ Median (5%, 95%) of failure probability distribution estimated for sample size re-estimation (SSRE) from 500 bootstrap re-samples. Based on first 278 patients in the current trial and 98 patients in the prior trial

^b^ Approximately the 20th percentile of the 500 failure-rate bootstrap re-samples. Based on first 278 patients in the current trial and 98 patients in the prior trial

**Fig. 1:**
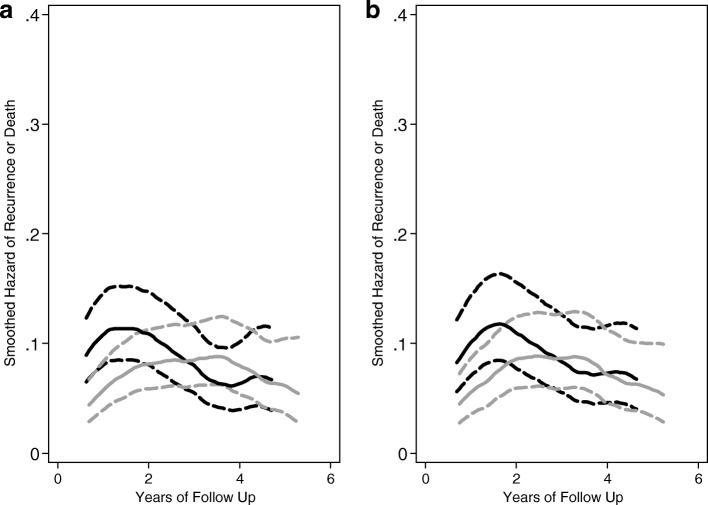
Hazard rate for recurrence or death for all randomized patients (**a**) and for the initial group of randomized patients (**b**). **a** All patients, *n* = 499, average follow up 4.2 years. **b** Initial group of randomized patients, *n* = 334, average follow up 4.6 years. Black line indicates the scheduled/luteal-phase-surgery group; gray line indicates the immediate/follicular-phase-surgery group. Dashed lines are pointwise 95% confidence intervals

**Fig. 2:**
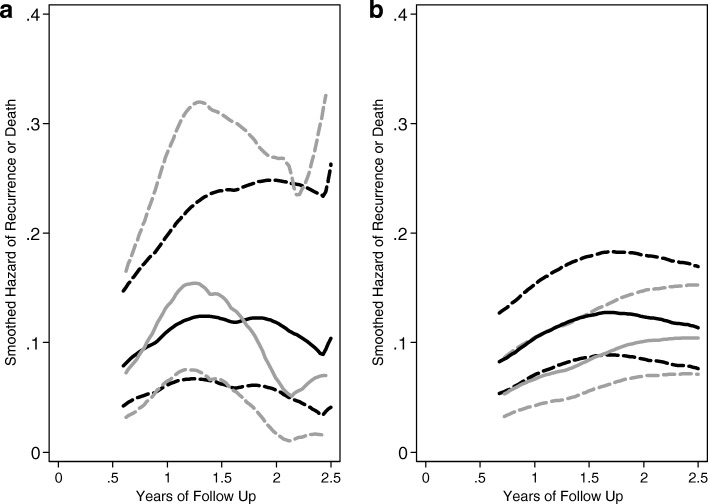
Hazard rate for recurrence or death for the sample size re-estimation (SSRE) cohort at the time of re-estimation (**a**) and through the final follow up (**b**). **a** At SSRE, *n* = 278, average follow up 1.2 years. **b** At final follow up, average follow up 4.2 years. Black line indicates the scheduled/luteal-phase-surgery group; gray line is for the immediate/follicular-phase-surgery group. Dashed lines are pointwise 95% confidence intervals

Investigating the hazard of failure over time illustrates some notable patterns and the inherent variability in hazard estimation at the time of SSRE. Figure 1 describes the hazard of failure by treatment group over time for all randomized participants and for those in the originally planned cohort. The general pattern and shape of these curves is consistent for the initial cohort and for the expanded one with full follow up. However, we note in Fig. 2 how variable the hazards were at the time of re-estimation, with markedly higher and more variable instantaneous failure probabilities. This variability was anticipated, and the re-estimation procedure accommodated this through supplementation of prior trial data and through re-sampling. By the end of study, these were far more smooth and stable for the re-estimation cohort.

Characteristics of patients at each site played a role in the failure probabilities obtained in the re-estimation timeframe. Table 3 describes patients who were observed in the re-estimation cohort and those recruited after re-estimation. Patients did not dramatically differ; however, more severe disease characteristics are notable in the SSRE cohort. Patients in the earlier cohort tended to more often be node positive. Moreover, 115 (42%) patients were recruited in the first period from site B and contributed 158.6 person years at SSRE, approximately half of all observed follow up time until this point in the trial (site A, 127.7 person years; other sites, 30.6 person years). These 115 patients from site B were more often of advanced stage (48% of patients with pathologic stage III-IV cancer, compared to 28% at site A) and had a higher rate of being node positive (67% of patients were node positive compared to 51% at site A). Over half (64%) of the observed failures at the time of re-estimation were from site B (site A, 29%; other sites: 7%). All other sites, beyond sites A and B, had only started to contribute patients and had little follow up time accrued at the time of re-estimation.

**Table 3:** Characteristics of patients contributing to SSRE and those recruited subsequently

	Included in SSRE (*n* = 272)^a^	Recruited after SSRE (*n* = 227)	95% CI for the difference
Age, years	42.1 (4.6)	42.5 (4.5)	−0.46 (− 1.27, 0.35)
Immediate/follicular-phase surgery	50.7% (138)	51.5% (117)	− 1.0% (− 9.6%, 8.0%)
Worse prognostic class^b^	22.1% (58)	19.0% (43)	3.0% (−4.1%, 10.2%)
Positive axillary nodes	60.5% (159)	55.3% (125)	5.1% (−3.6%, 13.9%)
Pathologic stage III-IV cancer	40.9% (110)	38.8% (88)	1.7% (−6.9%, 10.3%)
Radiotherapy given after mastectomy	40.2% (108)	38.8% (88)	1.4% (−7.2%, 10.0%)
Recruitment site			
A	34.6% (94)	33.5% (76)	1.1% (−7.3%, 9.4%)
B	42.3% (115)	44.1% (100)	−1.8% (−10.5%, 7.0%)
Other	23.2% (63)	22.5% (51)	0.7% (−6.7%, 8.1%)

^a^ Total of 499 of 509 of all randomized, 334 of 340 initial-cohort patients and 272 of 278 in the sample size re-estimation (SSRE) group were evaluable

^b^ Nodal status and pathologic stage available for 489 patients. Worse prognosis defined as having both positive axillary nodes and pathologic stage III-IV cancer

**Fig. 3:**
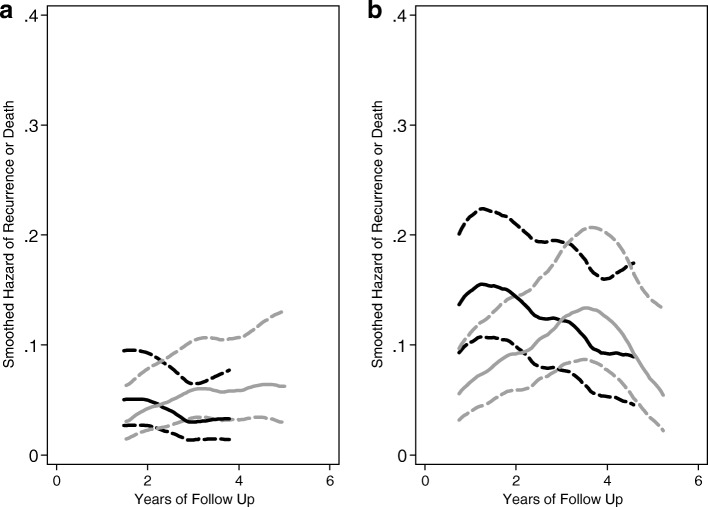
Hazard rate for recurrence or death for randomized patients at each major site of accrual at site A (**a**) and site B (**b**). **a** Site A, *n* = 170, average follow up 4.76 years. **b** Site B, *n* = 215, average follow up 4.0 years. Black line indicates the scheduled/luteal-phase-surgery group; gray line indicates the immediate/follicular-phase-surgery group. Dashed lines are pointwise 95% confidence intervals

Figure 3 illustrates the differential hazard of failure for the two major sites over the course of the entire follow up period. Site B had a higher overall failure experience for both treatment groups, likely due to the limited availability of radiation therapy as adjuvant treatment and a larger proportion of patients with later-stage disease at randomization. Radiation therapy was not part of the trial intervention but was often provided to patients at other sites as a part of standard care. Moreover, the failure experience at site A reveals that while patients receiving immediate/follicular-phase surgery had slightly lower, initial, instantaneous risks of failure compared to patients receiving scheduled/luteal-phase surgery, their risk increased over time, and hazard curves crossed just after 2 years of follow up. This crossing was not observed at site B until later in the follow up experience, after 3 years.

## Discussion

In the presented work, we review our experience from a blinded SSRE procedure for a time-to-event trial. Our circumstances, including the sensitive nature of the trial and not having a re-estimation procedure pre-specified in the study analysis plan, required that a blinded procedure be used to maintain study rigor and improve study design. We found that our re-estimation procedure did reasonably well in estimating the control-group failure probabilities at the time of re-estimation when the data were completely un-blinded and follow up was completed for the SSRE cohort. This was evidenced by observing the estimated (blinded) and actual (un-blinded) failure probabilities at the time of re-estimation. Further, as we had been conservative in utilizing information from the blinded SSRE procedure, sample size was increased only in response to effect size information that was available external to the current trial. Increasing sample size did provide more precise HR estimates, but did not change the overall inference from the trial. In fact, the anticipated treatment effect for scheduled luteal-phase surgery did not show beneficial impact on disease-free or overall survival [17].

Our SSRE procedure relied on two key assumptions. First, follow up data from a prior completed trial was used to supplement the follow up information not yet observed for the current trial participants. Participants’ data from the prior trial were included along with the SSRE cohort for the SSRE procedure. By including these prior participants’ data we assumed that they were reasonable proxies for information not yet observed in our current trial. Given that all prior trial participants were recruited from one of the current trial study sites, had the same disease diagnoses, and at the time of SSRE, prior and current study subjects had similar key prognostic characteristics such as nodal status, we were comfortable with this assumption. Second, we assumed that the characteristics of participants recruited over time would not substantially vary, particularly in prognostic characteristics.

An unanticipated benefit of detailed data examination and final analysis of the trial were observations on patient accrual and patient characteristics over the accrual period. This rigor was in part due to our careful examination of data to implement the procedure, and in part due to the sample size re-estimation being unplanned and our concern that the procedure would be viewed with skepticism. In our thorough examination, we found that patients accrued earlier tended to have somewhat more severe disease compared to those accrued later in time. At the time of re-estimation overall descriptive data suggested a larger proportion of higher-risk patients than was seen in the previous trial experience. Combined with patients accrued later, the comparison/control group (immediate/follicular-phase surgery) failure probabilities were estimated to be lower than were used in either the original trial design, and lower than the control-group failure probabilities estimated at the time of re-estimation. While a lower than anticipated failure function was observed in the comparison/control group, a higher than anticipated failure function was observed in the active-treatment group (schedule/luteal-phase surgery) resulting in a trial with no observed benefit of scheduled surgery. If the SSRE was planned for at the design stage, it would have been possible and, in retrospect, recommended, to incorporate either a formal interim analysis at the time of SSRE or a partially blinded SSRE procedure with a “gatekeeping”’ rule, requiring the DSMC to approve an increase of sample size only when the estimated treatment effect was greater than a defined minimum effect [25]. If in fact the DSMC had requested the un-blinded futility boundary at the time of sample size re-estimation, the trial would not have met a standard O’Brien-Fleming non-binding futility boundary. With 25% information at the time of re-estimation, the estimated HR was 1.32 (95% CI 0.63, 2.80, *p* value = 0.462), which is below the futility boundary of HR = 1.40, *p* value = 0.811) under the original design characteristics. However, either a formal interim analysis for futility or a rule for gatekeeping would have detected the increased failure rate in the active-treatment group.

Although the statistical and clinical trial literature has many examples of work to theoretically evaluate sample size re-estimation methods and performance through simulation, there have been few examples from real-world applications to detail the implementation and impact of adaptations, particularly from trials utilizing blinded sample size re-estimation [1, 2, 25]. McClure et al. provide a detailed review of their re-estimation procedure and trial results from a trial of secondary prevention of small subcortical stroke. The author’s re-estimation methods relied primarily on simulation studies, informed by the overall event rate in their current trial and extended follow up time, as well as published data on expected event probabilities for the time to recurrent stroke. No previous trial data were incorporated into their re-estimation procedure. Similar to our trial, they found that increasing sample size did not provide a clinically meaningful impact on final inference, beyond what would have been estimated by the initial trial design. Pritchett et al. report on a blinded SSRE as part of a more general group sequential design; however, no increase was made to their initial sample size estimate [25].

## Conclusions

Planning for minimal design modifications at the design stage, such as blinded (or partially blinded) sample size re-estimation, potentially coupled within a group sequential design, is well-accepted by regulatory and granting agencies [26]. Planning for an interim sample size increase requires (1) robust and reliable methods for SSRE, (2) expertise in these methods, and (3) the resources to eventually implement them, perhaps within some maximum allowable expansion. Pritchett et al. incorporated a gatekeeping rule with their blinded sample size re-estimation procedure, requiring that the DSMC approve an increase of sample size only when the estimated treatment effect was greater than a defined minimum effect [25]. Particularly for time-to-event outcomes, blinded procedures may be the best option to aid in maintaining power when combined with a gatekeeping or futility monitoring procedure to guard against unnecessary increases. In addition to the perception of reduced study integrity, it has been noted that there is an increased probability of un-blinded procedures recommending ineffective therapy [27]. When planned at initiation, blinded adaptations should provide efficiencies for resource allocation and in minimizing trial over-enrollment.

## Data Availability

The datasets used and/or analyzed during the current study are available from the corresponding author on reasonable request.

## Abbreviations

CI: Confidence interval
DSMC: Data Safety Monitoring Committee
HR: Hazard ratio
SSRE: Sample size re-estimation
